# In Vitro Effects of Charged and Zwitterionic Liposomes on Human Spermatozoa and Supplementation with Liposomes and Chlorogenic Acid during Sperm Freezing

**DOI:** 10.3390/cells13060542

**Published:** 2024-03-19

**Authors:** Elena Moretti, Claudia Bonechi, Cinzia Signorini, Roberta Corsaro, Lucia Micheli, Laura Liguori, Gabriele Centini, Giulia Collodel

**Affiliations:** 1 Department of Molecular and Developmental Medicine, University of Siena, 53100 Siena, Italy; cinzia.signorini@unisi.it (C.S.); r.corsaro@student.unisi.it (R.C.); laura.liguori@student.unisi.it (L.L.); gabriele.centini@unisi.it (G.C.); giulia.collodel@unisi.it (G.C.); 2 Department of Biotechnology, Chemistry and Pharmacy, University of Siena, 53100 Siena, Italy; claudia.bonechi@unisi.it; 3 Department of Medicine, Surgery and Neuroscience, University of Siena, 53100 Siena, Italy; lucia.micheli@unisi.it; 4 Obstetrics and Gynecological Clinic, University of Siena, 53100 Siena, Italy

**Keywords:** chlorogenic acid, human spermatozoa, liposomes, sperm freezing

## Abstract

Semen handling and cryopreservation induce oxidative stress that should be minimized. In this study, human semen was supplemented during cryopreservation with formulations of handmade liposomes and chlorogenic acid (CGA), an antioxidant compound. Zwitterionic (ZL), anionic (AL), and cationic (CL) liposomes were synthesized and characterized. Three aliquots of swim-up-selected sperm were incubated with ZL, AL, and CL (1:10,000), respectively. The percentages of sperm with progressive motility, high mitochondrial membrane potential (MMP; JC-1), double-stranded DNA (dsDNA acridine orange), and acrosome integrity (*Pisum sativum* agglutinin) were assessed. Then, human semen was frozen using both 1:10,000 ZL and CGA as follows: freezing medium/empty ZL (EL), freezing medium/empty ZL/CGA in the medium (CGA + EL), freezing medium/CGA loaded ZL (CGA), freezing medium (CTR). The same sperm endpoints were evaluated. ZL were the most tolerated and used for semen cryopreservation protocols. All the supplemented samples showed better endpoints versus CTR (*p* < 0.001). In particular, spermatozoa from the CGA and CGA + EL A samples showed increased motility, dsDNA, and acrosome integrity versus CTR and EL (*p* < 0.001; motility EL vs. CGA + EL *p* < 0.05). ZL and CGA can improve post-thaw sperm quality, acting on both cold shock effect management and oxidative stress. These findings open new perspectives on human and animal reproduction.

## 1. Introduction

Liposomes are spherical, biocompatible vesicles composed of one or more concentric phospholipid bilayers and enclosing an aqueous environment. These properties allow them to sequester both lipophilic and hydrophilic compounds in the lipid membrane and aqueous core, respectively.

Depending on the lipid composition, the liposome surface can be neutral, positively or negatively charged [1]. In in vitro studies, the mechanism and extent of interaction between liposomes and cells are strongly influenced by the type and density of liposome surface charge [2]. The literature on the applications of liposomes is extensive. Because liposomes are both non-toxic and biodegradable, they represent a powerful delivery system for various drugs and vaccines [3,4], and are also used in diagnostics and cosmetics [5]. Potential applications of liposomes include sperm research, as they physiologically interact with extracellular vesicles such as epididymosomes and prostasomes [6,7]. For these reasons, liposomes can be used in several areas of animal and human sperm research:

1. Cryopreservation: investigating the protective effect of liposomes on sperm damage due to the freezing protocols [8,9,10];
2. The study of membrane ion channels by the patch-clamp method, using sperm-derived liposomes [11];
3. Liposomes as vehicles of various compounds (e.g., ATP, antioxidants) [12,13,14,15];
4. Model systems to mimic and confirm biological phenomena occurring at the sperm membrane [16].

Collodel et al. [17], in an in vitro study using a model of pre-pubertal Sertoli cells, indicated zwitterionic liposomes, the same type used in this study, as the best carrier for drugs.

Recently, Luo et al. [18] used nasal administration of nerve growth factor-loaded liposomes to restore spermatogenesis in azoospermic mice; the authors concluded that this method could be considered a new potential therapy to activate the hypothalamic–pituitary–gonadal axis and increase the levels of androgenic hormones. The literature on the use of liposomes in human semen and spermatozoa is scant. Liposomes have been used in vitro as carriers of ATP [12], which triggered sperm capacitation, and quercetin, which increased DNA damage at a concentration of 100 µM [13]. In addition, Mutalik et al. [19] found that a freezing medium based on liposomes encapsulated with soy lecithin-cholesterol was a good alternative for cryopreservation protocols. The idea of loading antioxidants into liposomes and using them for cryopreservation is attractive. Many antioxidants have already been successfully used for media supplementation during cryopreservation of human semen. Freezing–thawing protocols enhance reactive oxygen species (ROS) production and, consequently, oxidative stress, which damages spermatozoa [20].

Chlorogenic acid (CGA) is an important and biologically active dietary polyphenol found in a variety of plants and in several foods, such as coffee, fruit, and tea. It displays different pharmacological activities, such as antioxidant, anti-inflammatory, anti-microbial, hepatoprotective, anticancer, anti-lipidemic, anti-diabetic, and other effects [21,22].

Chemically, CGA is a phenolic acid derived from the caffeic and quinic acids. Its molecular structure is responsible for antioxidant properties. CGA contains five hydroxyl groups and one carboxyl group. The phenolic hydroxyl groups can react with free radicals engaged in strong antioxidant activity [23].

In this study, three different lipid compositions with different surface charges (zwitterionic, cationic, and anionic) were used to synthesize liposomes, which were then incubated in vitro to evaluate their cytotoxicity to human spermatozoa, analyzing endpoints such as motility, DNA and acrosome status, and mitochondrial membrane potential (MMP). Then, zwitterionic liposomes loaded with CGA, a phenolic acid compound with antioxidant activity, were used as media supplements in a freezing–thawing procedure to investigate a possible effect in human sperm cryostability.

## 2. Materials and Methods

### 2.1. Liposome Preparation

DOPC (1,2-dioleoyl-sn-glycero-3-phosphocholine), DOPE (1,2-di-(9Z-octadecenoyl)-sn-glycero-3-phosphoethanolamine), DOPA (1,2-dioleoyl-sn-glycero-3-phosphate), and DOTAP (1,2-dioleoyl-3-trimethylammonium-propane) were purchased from Avanti Polar Lipids Inc., Alabaster, AL, USA. CGA was purchased from Sigma-Aldrich (St. Louis, MO, USA).

DOPC/DOPE (zwitterionic), DOPE/DOPA (anionic), and DOTAP/DOPE (cationic) liposomes were prepared at 1:0.5 molar ratio with a total lipid concentration of 1.0 × 10^−2^ M. The detailed protocol of preparation is reported in Collodel et al. [17].

In addition, CGA-loaded zwitterionic liposomes were prepared by rehydrating the phospholipid film with an aqueous solution of CGA (4 mg/mL), resulting in a multilamellar dispersion. Freeze–thaw cycles were carried out and liposomes were extruded (LiposoFast apparatus, Avestin, Ottawa, ON, Canada) using a 100 nm polycarbonate membrane. All liposomes were dialyzed using a dialysis membrane (molecular weight cut-off = 14.000) for 12 h. All liposomes were stored at 4 °C before subsequent analyses.

#### 2.1.1. Size and Surface Charge of Liposomes

The size and surface charge of the cationic, anionic, and zwitterionic empty liposomes and CGA-loaded zwitterionic liposomes were measured via dynamic light scattering (DLS, Zetasizer Nano ZS90, Malvern Instrument Ltd., Malvern, UK). The detailed procedure is described in Collodel et al. [17].

#### 2.1.2. Encapsulation Efficiency (EE) of Chlorogenic Acid

The EE% data were determined using UV-visible spectra (Perkin-Elmer Lamda 25 spectrophotometer, Waltham, MA, USA) at 25 °C. The detailed procedure is described in Collodel et al. [17]. A calibration curve was obtained by measuring the absorbance of the CGA solution at 330 nm.

### 2.2. Semen Samples

For this study, semen samples were obtained from 20 healthy volunteer donors (aged between 23 and 38 years) recruited at the Department of Molecular and Developmental Medicine, University of Siena (Siena, Italy). Donors were informed of the study protocol and their privacy was guaranteed.

Participants signed written informed consent forms before participating in this research, agreeing that their semen samples would be used for scientific purposes. The study was conducted in accordance with the Declaration of Helsinki, and the protocol was approved by the Ethic Committee Siena University Hospital (ID CEAVSE 25612).

Semen samples were collected by masturbation after 3–5 days of sexual abstinence and analyzed after liquefaction at 37 °C for 30 min. The basic semen analysis was performed following the World Health Organization (WHO) guidelines [24]. First, macroscopic characteristics (liquefaction, viscosity, volume, color, pH) were analyzed, then microscopic characteristics such as sperm concentration, motility, and morphology were assessed according to the WHO guidelines for semen analysis [24]. The morphology of the spermatozoa was evaluated with pre-stained Testsimplets^®^ slides (Waldeck GmbH & Co. KG, Münster, Germany).

#### Sperm Selection

To obtain the most homogeneous fraction of motile sperm possible, the swim-up technique was used. The technique was performed in four different sterile, conical tubes: A 0.5 mL semen sample was added to each tube and carefully overlaid with 0.5 mL of Sperm Washing Medium IrvineScientific^®^ (Santa Ana, CA, USA). The tubes were inclined at an angle of 45° and incubated at 37 °C for 45 min. Then, 0.5 mL of the uppermost medium, containing highly motile spermatozoa, was collected.

### 2.3. Use of Liposomes with Human Sperm Samples

**Figure 1:**
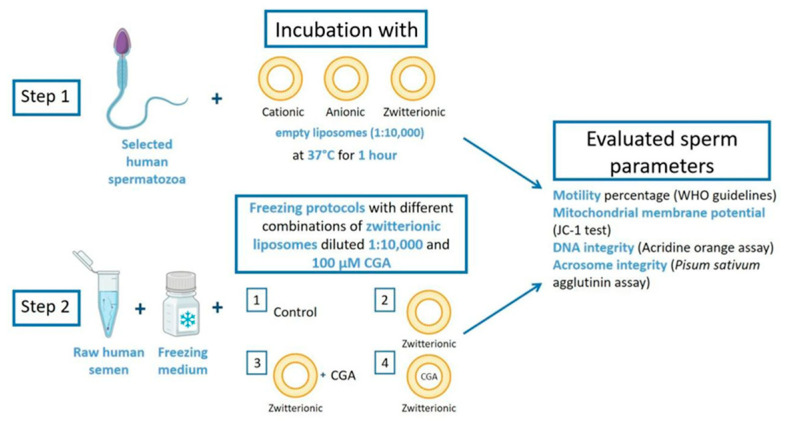
Graphical study design illustrating the two steps performed in this study. In step 1, selected spermatozoa were incubated with anionic, cationic, and zwitterionic liposomes, and endpoints such as motility, mitochondrial membrane potential, DNA and acrosome integrity were evaluated. In step 2, human semen samples were frozen, supplementing the medium with different combinations of zwitterionic liposomes and chlorogenic acid (CGA). The same endpoints were evaluated after thawing.

This research included two different steps (Figure 1):

Step 1 involved the incubation of selected spermatozoa with cationic, anionic, and zwitterionic empty liposomes to evaluate the effects on human sperm motility. The selected sperm were incubated with liposomes diluted 1:10,000 at 37 °C for 1 h. After incubation, the motility percentage was assessed according to WHO guidelines [24]. The other endpoints assessed were mitochondrial membrane potential (MMP, JC-1 test), DNA status (acridine orange assay), and acrosome integrity (*Pisum sativum* agglutinin assay, PSA).

Step 2 involved the use of zwitterionic liposomes and CGA as a supplement in semen freezing–thawing protocols. Basal semen was frozen under different conditions: (1) freezing medium alone (control, CTR); (2) freezing medium and zwitterionic empty liposomes diluted 1:10,000 (EL); (3) freezing medium with zwitterionic empty liposomes diluted 1:10,000 and 100 µM CGA (CGA + EL); and (4) freezing medium and CGA-loaded liposomes diluted 1:10,000 (CGA). The same endpoints were evaluated.

### 2.4. Evaluation of Sperm Motility

The sperm motility was evaluated according to the WHO guidelines [24]. Spermatozoa were analyzed at the light microscope level (objective 40× and ocular 10×, Leica Microsystems DM EP, Wetzlar, Germany) using a Burker counting chamber. The operator counted spermatozoa with rapid and slow progressive motility, and then non-progressive motility and immotile spermatozoa in ten fields of the Burker counting chamber. Rapid progressive motility indicates spermatozoa showing fast, linear active movement, while slow progressive motility indicates spermatozoa with a slow linear movement. Non-progressive motility includes spermatozoa with tail movements with an absence of progression. Finally, immotile spermatozoa are devoid of tail movement.

### 2.5. Evaluation of Sperm DNA Integrity by Acridine Orange (AO) Test

Acridine orange (AO) staining reveals the vulnerability of sperm DNA to acid-induced denaturation by highlighting the metachromatic shift of AO fluorescence from green (double-stranded [ds] DNA) to red (denatured, single-stranded [ss] DNA). AO emits green fluorescence when it is intercalated into native dsDNA as a monomer, and red when it binds to ssDNA as an aggregate. The method used was reported by Moretti et al. [13]. Briefly, a stock solution of 0.1% AO (3, 6-bis [dimethylamino] acridine, hemi [zinc chloride] salt, BDH Chemicals Ltd., Poole, UK) was prepared and stored in the dark at 4 °C until use.

Each treated sample was smeared onto pre-cleaned glass slide and fixed overnight in Carnoy fixative (methanol acetic acid 3:1). Then, slides were stained for 5 min with a working solution freshly prepared by mixing four parts AO stock solution with sixteen parts 0.1 M citrate and one part 0.3 M Na_2_HPO_4_ 7H_2_O.

Then, the slides were gently rinsed with distilled water, mounted, and immediately evaluated with a Leitz Aristoplan fluorescence microscope (Leica, Wetzlar, Germany) equipped with a 490 nm excitation light and a 530 nm barrier filter. Nuclei from 300 spermatozoa were examined and scored as green or red (sometimes orange yellow) depending on the fluorescence. The results were expressed as the percentage of the sperm with normal, double-stranded DNA (dsDNA, green fluorescence).

### 2.6. Evaluation of Sperm Mitochondrial Membrane Potential (MMP) by JC-1 Dye

Sperm MMP was determined using the dye 5,5′,6,6′-tetrachloro-1,1′,3,3′-tetraethylbenzimidazolecarbocyanine iodide (JC-1) (Molecular Probes, Eugene, OR, USA), as described in Noto et al. [25]. The stock solution of JC-1 at the concentration of 1 mg/mL in dimethylsulfoxide (DMSO) was prepared, divided into aliquots, and stored in a freezer at −20 °C until use. Briefly, sperm samples were incubated in the dark with 1 μg/mL JC-1 dye (diluted in PBS) for 20 min at 37 °C. Then, the samples were centrifuged at 400× *g* for 10 min, resuspended in PBS, and smeared onto glass slides.

Slides were evaluated using a Leitz Aristoplan fluorescence microscope (Leica, Wetzlar, Germany) equipped with a 490 nm excitation light and a 530 nm barrier filter. The red-stained midpiece, due to the presence of JC-1 aggregates, indicated a high MMP, whereas the midpiece with a diffuse green signal (monomeric form of JC-1) indicated low MMP. At least 300 sperm were evaluated at 1000× magnification. The results were expressed as percentage of sperm with high MMP.

### 2.7. Evaluation of Sperm Acrosome Integrity Using Pisum Sativum Agglutinin (PSA)

PSA is a lectin that binds the carbohydrate regions of glycoproteins, particularly those located in the acrosome. Sperm samples were washed with PBS, centrifuged at 400× *g* for 10 min, resuspended in PBS, and smeared onto glass slides. The slides were fixed in methanol for 20 min and acetone for 5 min at −20 °C, air-dried, and frozen at −20 °C until use. The slides, thawed and washed twice in PBS for 5 min, were treated for 30 min at room temperature with tetramethylrhodamine (TRITC)-conjugated PSA (PSA, 21761046, Genelinx International Inc., dba bio WORLD, Dublin, Ireland) diluted 1:1000 in PBS. After rinsing in PBS for 15 min, the slides were treated with 4′,6-diamidin-2-fenilindolo (DAPI) in PBS for 10 min at room temperature to stain the nuclei. The slides were observed and evaluated with a Leitz Aristoplan fluorescence microscope (Leica, Wetzlar, Germany). The spermatozoa with intact acrosomes showed intense red fluorescence in the acrosomes, while an absence of fluorescence or staining in the equatorial region indicated sperm with reacted acrosomes.

### 2.8. Freezing–Thawing Protocol Using Chlorogenic Acid and Zwitterionic Liposomes as Supplement

Each basal semen sample was divided into four aliquots, and the cryopreservation medium (Test Yolk Buffer with gentamicin sulphate; FujiFilm, Irvine Scientific, Santa Ana, CA, USA) was added dropwise in a 1:1 ratio (vol:vol) and gently mixed. Then, the semen aliquots were treated as follow:

(1) 1:10,000 empty zwitterionic liposomes (EL);
(2) 1:10,000 empty zwitterionic liposomes and 100 µM CGA (CGA + EL);
(3) 1:10,000 CGA-loaded zwitterionic liposomes (CGA);
(4) The untreated aliquot with semen and cryopreservation medium represented the control (CTR).

Samples were transferred into cryovials with final volumes of 0.3 mL. Cryovials were stored at 4 °C for 30 min and for 1 h at −20 °C, and then immersed in liquid nitrogen at −196 °C.

Two weeks later, the cryovials were thawed at 37 °C for 10 min. The sperm motility percentage was determined according to WHO guidelines [24]. Then, the specimens were centrifuged at 400× *g*, and spermatozoa were examined for DNA integrity, MMP, and acrosome shape, as previously described. Ten semen samples were used for this experiment.

### 2.9. Statistics

Statistical analysis was carried out using the SPSS software package, version 17.0, for Windows (SPSS Inc., Chicago, IL, USA). The normality of the variable distributions was verified with the Kolmogorov–Smirnov test. Since non-parametric distribution was observed, specific non-parametric tests were applied. The Kruskal–Wallis test was used to compare the differences among different groups in step one, when selected spermatozoa were treated with the 3 type of liposomes, and in step two, when human semen was frozen with liposomes and CGA used in different combinations (control [CTR], empty liposomes [EL], CGA-loaded liposomes [CGA], empty liposome + CGA in the medium [CGA + EL]). When a significant difference was found, Tukey’s post hoc test was applied for pairwise post hoc testing. Data were reported as medians (interquartile range [IQR]). *p* < 0.05 was considered significant.

## 3. Results

The dynamic light scattering (DLS) results obtained for the physico-chemical characterization of liposomes are reported in Table 1. The DLS experiments were performed after the purification processes.

The mean diameter of the synthesized liposomes was consistent with the extrusion process using polycarbonate membranes with 100 nm pores. The different chemical structures and charges of the phospholipids did not change the sizes of the vesicles. The DOPC/DOPE + CGA liposomes showed an increase in mean diameter due to the loading process with the water-soluble drug, which changed their geometry. The low polydispersity indexes (P.I.) showed by all the systems revealed that the liposomes were monodisperse, and the incorporation of CGA did not alter the physical stability of the zwitterionic vesicles.

Analyzing the data reported in Table 1, it can be observed that DOPC/DOPE liposomes had a negative ζ-potential, though the net polar head charge of zwitterionic phospholipids was zero. The encapsulation of CGA did not change the value of the surface charge. The ζ-potential allowed for evaluation of the aggregation processes in the solution. It is well known that systems with ζ-potentials more positive than +30 mV or more negative than −30 mV are usually considered stable. This information suggested that the cationic and anionic liposomes were the most stable over time by presenting less aggregation processes in solution than the zwitterionic liposomes.

As shown in Table 1, the UV-visible analyses revealed an encapsulation efficiency of 68.4 ± 5.9%. The liposome preparation procedure used in the present work resulted in a relatively high incorporation rate for CGA, indicating that the zwitterionic DOPC/DOPE liposomes are good encapsulation systems for water-soluble drugs.

### 3.1. Semen Parameters of Donors

The WHO guidelines [24] were used as reference for semen analysis. All samples showed sperm parameters in the normal range: sperm concentration/mL, progressive sperm motility %, and normal sperm morphology % were between the 25th and 75th percentiles, and the vitality % was between the 50th and 75th percentiles.

### 3.2. Step 1: Treatment of Selected Human Sperm with Cationic, Anionic, and Zwitterionic Liposomes

After incubation of selected human spermatozoa with cationic, anionic, and zwitterionic liposomes, the percentage of progressive motility, the percentage of sperm with dsDNA, the percentage of sperm with high MMP, and the percentage of sperm with normal acrosomes were assessed, and the results are reported in Table 2.

Human spermatozoa incubated with cationic liposomes showed a significant increase in progressive motility (*p* < 0.05, Table 2) compared to that observed in control samples and in spermatozoa incubated with zwitterionic liposomes. The motility of spermatozoa incubated with both charged liposomes appeared to be hyperactivated, with asymmetric flagellar beating and head displacement, and this was particularly evident when cationic liposomes were used.

Spermatozoa treated with cationic liposomes showed a significantly higher DNA susceptibility to damage than that observed in spermatozoa incubated with anionic and zwitterionic liposomes and in untreated spermatozoa (*p* < 0.001, Table 2).

Another assessed endpoint was the MMP by means of the JC-1 probe, which differentiates between sperm with low (green fluorescence) and high (red fluorescence) MMP. The selected human sperm treated with cationic liposomes showed significantly increased MMP than the control samples (*p* < 0.05, Table 2). In addition, the percentage of sperm with high MMP was increased, although non-significantly, in samples treated with anionic liposomes. Finally, the acrosome shape was evaluated with TRITC-conjugated PSA. The percentages of spermatozoa with intact acrosomes after treatment with cationic, anionic, zwitterionic liposomes and controls were similar, as reported in Table 2.

For the cryopreservation experiment (step 2), zwitterionic liposomes were chosen because they did not alter motility characteristics, DNA status, or MMP. Their tolerability was also tested and observed in other type of cells [17,26].

### 3.3. Step 2: Freezing Spermatozoa with Different Combinations of Zwitterionic Liposomes and Chlorogenic Acid

**Figure 2:**
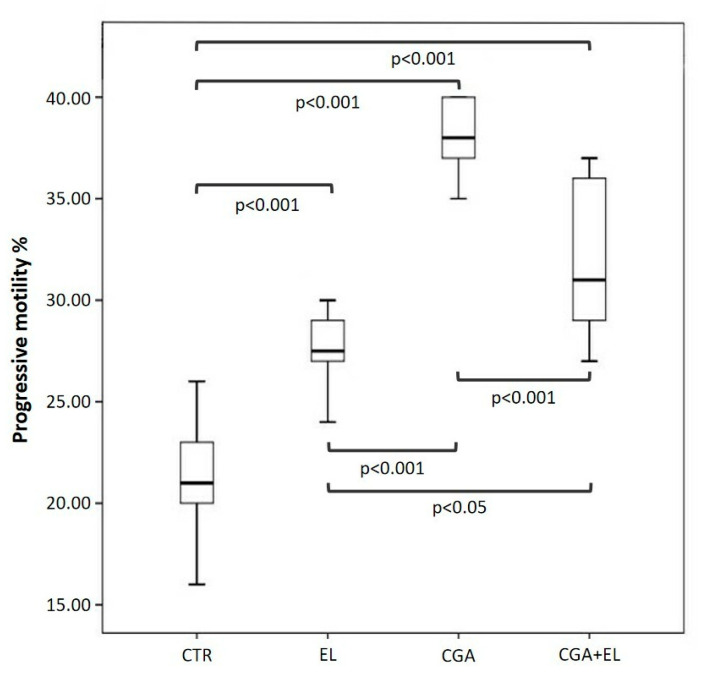
Median (IQR) of progressive motility percentage evaluated in frozen–thawed spermatozoa treated as follows: sperm frozen with cryopreservation medium (CTR); sperm frozen with cryopreservation medium and empty liposomes diluted 1:10,000 (EL); sperm frozen with cryopreservation medium and CGA-loaded liposomes (CGA); and sperm frozen with cryopreservation medium, empty liposomes diluted 1:10,000, and 100 µM CGA in the medium (CGA + EL).

**Figure 3:**
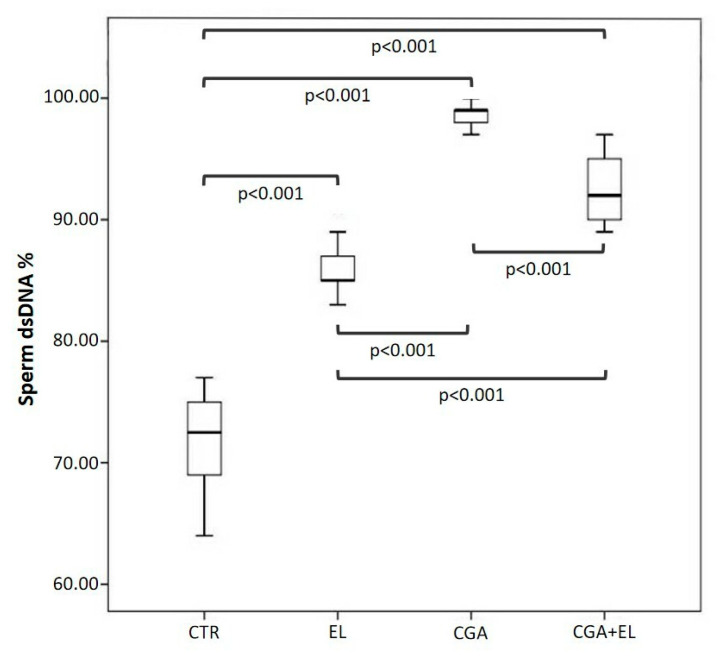
Median (IQR) of the percentage of sperm with double-stranded DNA (dsDNA) evaluated in frozen–thawed samples. The samples were treated as follows: sperm frozen with cryopreservation medium (CTR); sperm frozen with cryopreservation medium and empty liposomes diluted 1:10,000 (EL); sperm frozen with cryopreservation medium and CGA-loaded liposomes (CGA); and sperm frozen with cryopreservation medium, empty liposomes diluted 1:10,000, and 100 µM CGA in the medium (CGA + EL).

**Figure 4:**
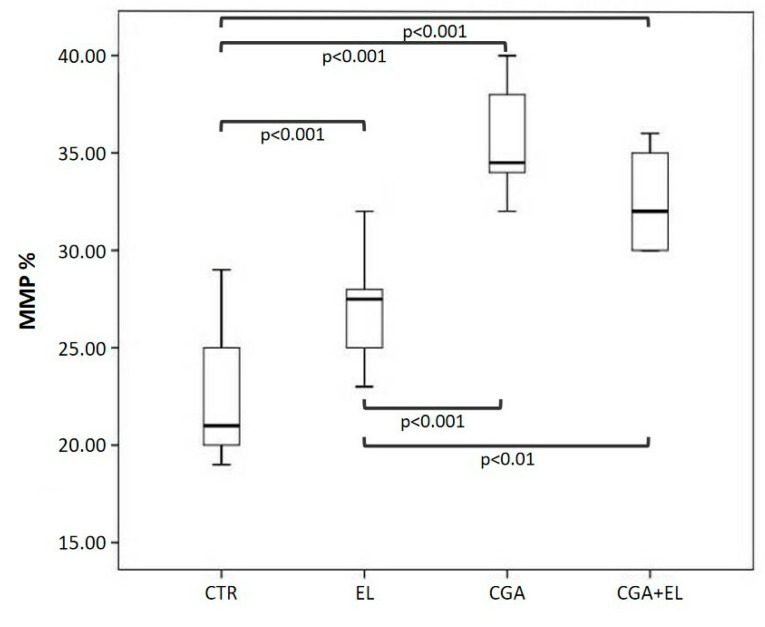
Median (IQR) of the percentage of sperm with high mitochondrial membrane potential (MMP) evaluated in frozen–thawed samples. The samples were treated as follows: sperm frozen with cryopreservation medium (CTR); sperm frozen with cryopreservation medium and empty liposomes diluted 1:10,000 (EL); sperm frozen with cryopreservation medium and CGA-loaded liposomes (CGA); and sperm frozen with cryopreservation medium, empty liposomes diluted 1:10,000, and 100 µM CGA in the medium (CGA + EL).

Each basal semen sample was divided into four aliquots: a control (CTR), a sample treated with 1:10,000 empty liposomes (EL), a sample treated with CGA-loaded liposomes (CGA) diluted 1:10,000, and a sample treated with empty liposomes and 100 µM CGA added to the cryopreservation medium (CGA + EL). The aliquots were frozen and thawed after 2 weeks as reported in the Materials and Methods section. The percentages of sperm with progressive motility, dsDNA, high MMP, and normal acrosomes were assessed after thawing, and the results are reported in Figure 2, Figure 3 and Figure 4.

The percentage of sperm with progressive motility (Figure 2) was significantly higher in all samples cryopreserved with zwitterionic liposomes (EL: 27.5 [26.2–29.2], CGA: 38.0 [36.5–40.0], CGA + EL: 31.0 [28.7–36.0]) compared to that of the control group (CTR: 21.0 [19.7–23.5]; *p* < 0.001).

The sperm motility of the samples incubated with EL was significantly reduced with respect to that measured in the CGA (*p* < 0.001) and CGA + EL (*p* < 0.05) samples, and the same parameter was higher in spermatozoa treated with CGA-loaded liposomes than that observed in spermatozoa incubated with CGA + EL (*p* < 0.001).

The percentage of sperm with dsDNA was significantly higher in frozen–thawed spermatozoa treated with zwitterionic liposomes, both empty and with CGA (EL: 85.0 [84.5–87.5], CGA: 99.0 [97.7–99.0], CGA + EL: 92.0 [89.7–95.0]), compared to that of the control group (CTR: 72.5 [68.0–75.3]; *p* < 0.001). Also, for this endpoint, the best results were obtained in the CGA group (Figure 3).

In all samples treated with zwitterionic liposomes (EL: 27.5 [24.7–29.0], CGA: 34.5 [33.7–38.0], CGA + EL: 32.0 [30.0–35.2]), we observed a significant increase in spermatozoa with high MMP with respect to control group (CTR: 21.0 [20.0–25.2]; *p* < 0.001, Figure 4).

The percentage of spermatozoa with high MMP was significantly lower in the EL group than that observed in the CGA and CGA + EL samples (*p* < 0.001 and *p* < 0.01, respectively).

**Figure 5:**
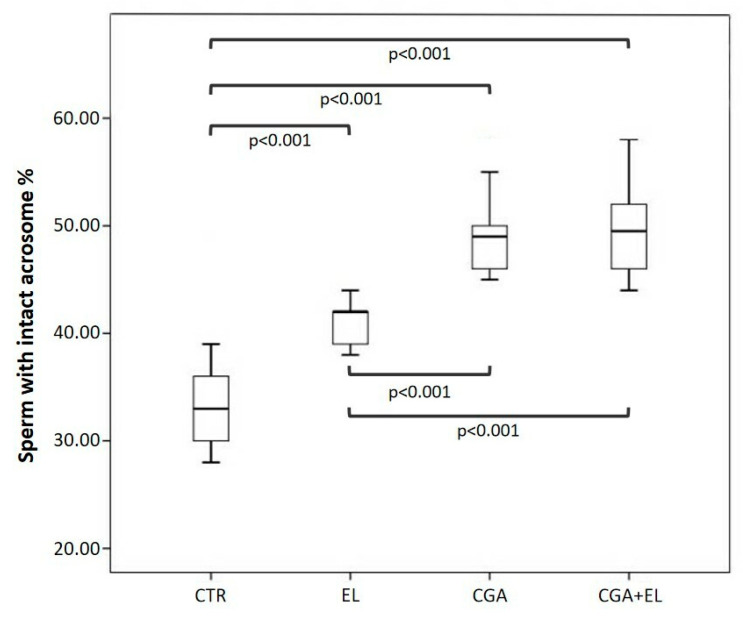
Median (IQR) of the percentage of sperm with intact acrosomes (TRITC-conjugated PSA) evaluated in frozen–thawed samples. The samples were treated as follows: sperm frozen with cryopreservation medium (CTR); sperm frozen with cryopreservation medium and empty liposomes diluted 1:10,000 (EL); sperm frozen with cryopreservation medium and CGA-loaded liposomes (CGA); and sperm frozen with cryopreservation medium, empty liposomes diluted 1:10,000, and 100 µM CGA in the medium (CGA + EL).

Finally, all samples treated with zwitterionic liposomes (EL: 42.0 [39.0–42.5], CGA: 49.0 [46.0–51.0], CGA + EL: 49.5 [45.7–53.0]) showed significant increases in the percentage of sperm with normal acrosomes with respect to control group (CRT: 33.0 [29.7–36.2]; *p* < 0.001). The acrosome protection was particularly evident in spermatozoa supplemented with CGA, either loaded in the liposomes or added in the cryopreservation medium, with respect to EL group (Figure 5).

**Figure 6:**
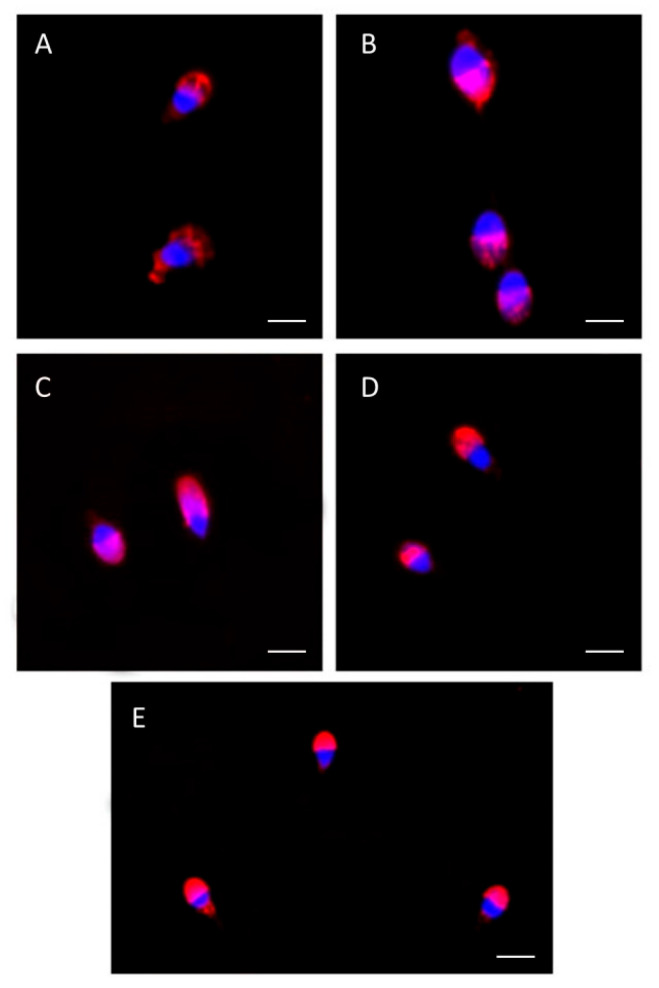
UV micrographs of human spermatozoa treated with TRITC-conjugated PSA. (**A**) shows frozen–thawed human spermatozoa used as the control, with altered acrosomes. Some acrosomal alterations are also visible in the frozen–thawed human spermatozoa treated with zwitterionic empty liposomes (**B**). The acrosomes appeared normal in samples supplemented with chlorogenic acid, both when loaded in zwitterionic liposomes (**C**) and when added to the medium with zwitterionic empty liposomes (**D**). Human sperm from a basal semen sample treated with TRITC- conjugated PSA is shown (**E**). Bars: (**A**,**C**,**D**) 6 µm; (**B**) 4 µm; (**E**) 8 µm.

Figure 6 shows the frozen–thawed human spermatozoa used as the control (Figure 6A), the group treated with zwitterionic empty liposomes (Figure 6B), the group treated with CGA-loaded zwitterionic liposomes (Figure 6C), and the group treated with zwitterionic empty liposomes and CGA added to the medium (Figure 6D). A UV micrograph showing human sperm from a basal sample treated with TRITC-conjugated PSA (Figure 6E) is also shown.

## 4. Discussion

Liposomes are man-made membrane vesicles used for many purposes [27]. This research comprises two steps. The first one was aimed at studying the in vitro effects of positively and negatively charged and zwitterionic liposomes on human sperm. The study of liposomes with different superficial charges enables the evaluation of chemical and physical properties that can affect both the encapsulation and the effects on different types of cells [26]. One issue to consider concerns the interaction between charged or zwitterionic liposomes and human spermatozoa. Garrett et al. [28], using liposomes loaded with membrane-impermeable agents, reported that these vesicles interact with spermatozoa; in addition, when loaded with messengers, such as calcium ions, the liposomes were proposed as a useful tool for investigating the cell signaling linked to sperm activation. Many other literature papers have reported interactions between loaded liposomes and spermatozoa, which have simply analyzed the effect of the loaded compound [12,13,29].

To accomplish the first step of this research, human spermatozoa were incubated with empty liposomes (cationic, anionic, and zwitterionic), and endpoints such as motility MMP, DNA, and acrosome integrity were evaluated. This protocol considered the investigation of the main sperm characteristics and structures, giving a general outcome of the sperm’s response to the treatments [20]. The experiments were carried out in a selected sperm population which was as homogenous as possible, where it was easier to detect the possible changes caused by the interaction with liposomes. Although, at the concentration used, the anionic and zwitterionic liposomes were not toxic for human spermatozoa, zwitterionic liposomes were the most tolerated ones and, for this reason, they were preferred for the other experiments. Mammalian sperm plasma membranes are mainly composed of phosphatidylcholine and phosphatidylethanolamine [30], which represent the phospholipids present in the zwitterionic liposomes.

On the contrary, spermatozoa incubated with cationic liposomes showed an increased, hyperactivated-like sperm motility characterized by high amplitude and asymmetric flagellar bends [31]. This observation, along with the increased percentage of sperm with high MMP (JC-1) and sperm DNA susceptibility to damage (AO test), suggested that cationic liposomes could induce an initial step of capacitation, a process necessary for fertilization that occurs physiologically near the oocyte. The acrosome was intact in all samples, and the liposome treatments did not cause an increase in acrosomal reaction. During capacitation, spermatozoa undergo several changes, such as hyperactivated motility, sperm–zona pellucida recognition, and, finally, acrosome reaction. Despite this process having been described more than half a century ago, the molecular mechanisms underpinning capacitation are not completely understood [32]. Regarding the observed sperm DNA’s susceptibility to damage, several studies have reported genotoxicity of cationic liposomes in vitro, and an increase in surface charge has been correlated with an increase in genotoxicity. The cationic head group probably induces ROS generation, causing inflammation. Cationic liposomes, with their positively charged surfaces, enable the particles to cross the cell membrane, interfering with DNA integrity [33,34]. It is interesting that Simon et al. [35] demonstrated, through micro-electrophoresis, that positively charged sperm have a higher percentage of DNA damage than negatively charged sperm, which conceivably represent the mature sperm sub-population. These results can support our observations on the relationship between cationic liposomes and decreased sperm DNA integrity.

In addition, amplified mitochondrial activity, observed in spermatozoa incubated with cationic liposomes, is a known marker of sperm capacitation, and several lines of evidence indicate that regulated calcium entry into mitochondria increases the efficiency of oxidative respiration [36]. For all these reasons, cationic liposomes were not considered for freezing media supplementation.

In the second step of this study, the well-tolerated zwitterionic liposomes were loaded with CGA and used as a supplement for freezing media during human spermatozoa cryopreservation. Similar zwitterionic liposomes have been revealed to be non-toxic and well tolerated in experiments with porcine Sertoli cells [17] and fibroblasts [26].

Recently, some of our group observed that CGA exerted a protective activity on human spermatozoa in cryopreservation protocols [25]. Because of the well-known behavior of CGA with human spermatozoa, we decided to load zwitterionic liposomes with this antioxidant molecule.

The cryopreservation of human semen plays a significant role in reproductive medicine and fertility preservation; it consists of freezing spermatozoa and storing them in liquid nitrogen at −196 °C. The storage at such a low temperature ensures very low thermal energy, and the cell metabolism slows down [37,38]. In animals, semen cryopreservation is even more common and contributes to genetic improvement through artificial insemination, as well as supporting the preservation of endangered species and the conservation of biodiversity [10]. Spermatozoa, compared to other cells, could be potentially perfect candidates for cryobiology because they are small cells with a large surface area (without cytoplasm in normal conditions); however, the harmful effects of cryopreservation on sperm function are well known [39].

The inability of cryopreservation media to totally prevent sperm from damage during freezing is one of the issues to be considered; as a result, frozen sperm have a reduced capacity for fertilization compared to fresh samples. The main forms of sperm damage caused by freezing–thawing procedures affect the membrane integrity, the cytoskeleton, the mitochondrial activity, the motility, the vitality, and the DNA fragmentation [38,40,41].

Several variables have been proposed as causes of poor post-thaw sperm quality, including sudden temperature fluctuations; ice crystal formation; osmotic stress; and higher generation of ROS, which causes oxidative insults. Overproduction of ROS is known to affect the sperm’s function and genome stability.

Different strategies have been proposed to reduce cryoinjuries in spermatozoa, including the use of different cryoprotectants, antifreeze proteins, and glycoproteins, as well as the addition of antioxidants to media, and even the application of mild stress before freezing in order to induce an adaptive reaction of spermatozoa to show a better post-thawing quality [42].

Many studies have focused on the use of liposomes as cryoprotectant additives during the cryopreservation of animal semen to limit the freezing–thawing-induced damages and improve breeding aims. For this purpose, it can be mentioned that a commercial extender based on liposomes (OptiXcell^®^) is available, and it is used in the cryopreservation of sperm from several animal species [10,43].

On the contrary, very little data on human semen are present in the literature [19].

The results obtained in this research indicated that the supplementation of a cryopreservation medium with all liposome formulations (empty liposome, CGA-loaded liposomes, CGA + empty liposomes) increased the cryostability and the functional competence of human spermatozoa. Purdy and Graham [44] reported that the transition of lipids to the gel phase during cooling and freezing is highly dependent on the lipid composition of the membranes; therefore, liposome interaction facilitates the membrane repair and a rearrangement of cell membrane components by modifying the membrane’s physicochemical properties, thereby improving the cryostability of the spermatozoa. Therefore, the improvement observed in samples supplemented with empty liposomes could be explained by the fact that the liposomes can act as excellent reservoirs of phospholipids and help in replenishing those lipids that are lost from the sperm membrane damaged by cold shock during the freezing process.

**Figure 7:**
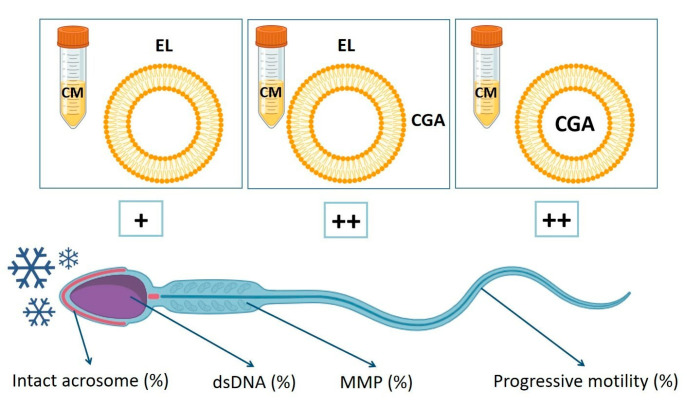
Graphical summary of the effects of zwitterionic liposomes and chlorogenic acid (CGA), used in different formulations, on human sperm endpoints during the freezing–thawing process. The left square indicates the sperm samples frozen with cryopreservation medium (CM) supplemented with empty liposomes (EL); the square in the middle indicates the sperm samples frozen with CM supplemented with both EL and CGA in the medium; the square on the right indicates the sperm samples frozen with CM supplemented with CGA-loaded liposomes. The symbols “+” and “++” indicate the positive effects of the different supplementations on the percentages of sperm with progressive motility, with double stranded (ds) DNA, with intact acrosomes, and with high mitochondrial membrane potential (MMP). Even if EL is protective for human sperm (+), the best results were obtained when liposomes and CGA, either loaded (++) in the liposomes or free in the medium (++), were used together.

In addition, our data indicate that the concomitant use of CGA, both loaded in the liposomes and free in the media, resulted in a significant increase in the sperm’s progressive motility, DNA integrity, mitochondrial activity, and acrosome stability (Figure 7).

Noto et al. [25] demonstrated the cryo-protective and antioxidant activity of CGA on human spermatozoa. Among the considered endpoints, DNA integrity was the most responsive one, and this was in accord with the results of the present research. This observation is important considering that spermatozoa are cells with very low transcriptional activity and repairing mechanisms, making them vulnerable to ROS, which are over-produced during cryopreservation. Noto et al. [25] suggested that the protective activity of CGA on spermatozoa during freezing–thawing protocols could also be mediated by a mechanism involving the purinergic system [45] that has been demonstrated to be present in human spermatozoa [46].

## 5. Conclusions

In conclusion, zwitterionic DOPC/DOPE liposomes can represent an excellent drug delivery system for a water-soluble drug. In addition, they are compatible with human spermatozoa, and, when used in combination with CGA, can improve the post-thaw sperm quality, acting both on the management of cold shock effects and on ROS generation. These findings can open a new field of studies with a large number of perspectives on human and animal reproduction.

## Figures and Tables

**Table 1:** Mean size distribution with polydispersity index (P.I.) and ζ-potential for all types of liposomes. Encapsulation efficiency of chlorogenic acid (CGA) into zwitterionic liposomes is also shown. The values are the averages of three measurements (mean ± SD).

Liposome Composition	Mean Diameter (nm) ± SD	P.I.	*ζ*-Potential (mV) ± SD	Encapsulation Efficiency (EE%)
DOPC/DOPE (1:0.5)	116.5 ± 9.2	0.11	−18.78 ± 4.22	
DOPC/DOPE + CGA	146.9 ± 9.4	0.16	−17.54 ± 2.29	68.4 ± 5.9
DOPC/DOTAP (1:0.5)	147.5 ±2.1	0.16	61.00 ± 1.50	
DOPE/DOPA (1:0.5)	148.3 ± 8.3	0.14	−38.28 ± 2.72	

**Table 2:** Median [IQR] percentage of swim-up selected spermatozoa with progressive motility, dsDNA, high MMP, and normal acrosomes in the four analyzed groups.

	Control (Ct)	Cationic Liposomes (C)	Anionic Liposomes (A)	Zwitterionic Liposomes (Z)	Kruskal–Wallis Test	Tukey’s Post Hoc Test
Progressive motility %	80.5 [74.2–81.0]	84.0 [82–86.2]	81.5 [78.5–84.0]	79.5 [74.5–80.5]	*p* < 0.05	Ct vs. C *p* < 0.05 C vs. Z *p* < 0.05
Sperm dsDNA %	97.5 [96.2–98.5]	82.5 [78.7–85.2]	97.5 [96.7–100]	97.0 [94.7–99.3]	*p* < 0.001	C vs. Ct *p* < 0.001 C vs. A *p* < 0.001 C vs. Z *p* < 0.001
Sperm high MMP %	62.0 [59.0–66.2]	72.5 [67.7–75.2]	68.0 [62.2–69.2]	64.5 [59.7–67.0.2]	*p* < 0.05	Ct vs. C *p* < 0.05
Normal acrosome %	84.0 [81.5–87.0]	80.0 [80.0–84.5]	84.5 [80.0–87.8]	85.0 [81.0–86.5]	Non-significant	

## Data Availability

The data generated and analyzed during this study are included on this published article and are available from the corresponding author.
